# Supplementary material for: Selection of Bacterial Strains for Control of Root-Knot Disease Caused by Meloidogyne incognita
Source: Microorganisms. 2021 Aug 10;9(8):1698. doi: 10.3390/microorganisms9081698 (PMC8402187; doi:10.3390/microorganisms9081698)
Supplement: Supplementary file 1 [file microorganisms-09-01698-s001.zip › microorganisms-1301210-supplementary.pdf]

Table S1. Bacterial strains collected from Krasnodar region used in this study.

Strain [1]	Collection Location (District)	Origin
BZR 18	Zernogradsky	winter wheat rhizosphere
BZR 59	Zernogradsky	winter wheat rhizosphere
BZR 86	Krylovskoi	winter wheat rhizosphere
BZR 148	FSBSI	winter oilseed rape rhizosphere
BZR 187	FSBSI	winter oilseed rape phylloplane
BZR 241	FSBSI	winter oilseed rape phylloplane
BZR 261	FSBSI	winter oilseed rape rhizoplane
BZR 277	FSBSI	winter oilseed rape rhizosphere
BZR 337	Krylovskoi	winter wheat rhizoplane
BZR 348	Krylovskoi	winter oilseed rape rhizosphere
BZR 367	Krylovskoi	winter wheat rhizoplane
BZR 413	Zelinsky	winter oilseed rape rhizosphere
BZR 416	Eysky	winter wheat rhizoplane
BZR 417	Eysky	winter wheat rhizoplane
BZR 430	Pavlovsky	winter wheat rhizoplane
BZR 436	Leningradsky	winter wheat rhizoplane
BZR 441	Zelinsky	winter wheat rhizosphere
BZR 455	Vyselkovsky	winter wheat rhizosphere
BZR 462	Vyselkovsky	winter wheat rhizosphere
BZR 472	Tcherbinovsky	winter wheat rhizosphere
BZR 480	Eysky	winter wheat rhizosphere
BZR 512	Gulkevichsky	winter wheat rhizoplane
BZR 517	Pavlovsky	winter wheat rhizoplane
BZR 519	Pavlovsky	winter wheat rhizoplane
BZR 523-1	Gulkevichsky	winter wheat rhizosphere
BZR 523-2	Gulkevichsky	bulk soil, soybean
BZR 528	Vyselkovsky	winter wheat rhizosphere
BZR 538	Zelinsky	winter wheat rhizosphere
BZR 623	Gulkevichsky	winter wheat rhizosphere
BZR 658	Pavlovsky	winter wheat rhizoplane
BZR 673	Krylovskoi	winter wheat rhizosphere
BZR 854	FSBSI	bulk soil, soybean
BZR 862	FSBSI	bulk soil, soybean
BZR 873	FSBSI	soybean rhizoplane

[1] Accession number of bacteria in the Bioresource Collection "State Collection of Entomoacariphages and Microorganisms" of Federal Scientific Center of Biological Plant Protection (Bioresource Collection of FSCBPP).

Table S2. Enzymatic activity of bacterial strains from the Bioresource Collection of FSCBPP.

Strain [1]	Lypase Activity (Day)				Chitinolytic Activity (Day)				Proteolytic Activity (Day)			
	4	8	14	20	4	8	14	20	4	8	14	20
BZR 18												
BZR 59										+	++	++
BZR 86								+				
BZR 148												
BZR 187	+	++	+++	++++					+	++	+++	++++
BZR 241									+	++	+++	++++
BZR 261												
BZR 277		+	++	+++					+	++	+++	++++
BZR 337	+	+	++	+++					+	++	+++	++++
BZR 348			+	++					+	++	+++	++++
BZR 367	+	+	++	+++					+	++	+++	++++
BZR 413	+	++	+++	++++				+	+	++	+++	+++
BZR 416	+	++	+++	++++					+	++	+++	+++
BZR 417	+	++	+++	++++								
BZR 430									+	++	+++	+++
BZR 436	+	++	+++	++++						+	++	++
BZR 441	+	++	+++	++++					+	++	+++	+++
BZR 455	+	++	+++	++++								
BZR 462						+	++	+++	+	++	+++	+++
BZR 472	+	++	+++	+++					+	++	+++	+++
BZR 480		+	++	+++					+	++	+++	+++
BZR 512							+	++	+	++	+++	+++
BZR 517									+	++	+++	+++
BZR 519									+	++	+++	+++
BZR 523-1		+	++	+++						+	++	++
BZR 523-2		+	++	+++						+	++	++
BZR 528										+	++	++
BZR 538		+	++	+++					+	++	+++	+++
BZR 623	+	++	+++	++++					+	++	+++	++++
BZR 658	+	++	+++	++++		+	++	+++	+	++	+++	++++
BZR 673							+	++	+	++	++	+++
BZR 854												
BZR 862							+	++				
BZR 873									+	++	+++	++++

[1]Accession number of bacteria in the Bioresource Collection of FSCBPP; + = very weak activity; ++ = weak activity; +++ = moderate activity; ++++ = strong activity

Table S3. Effect of bacterial strains on growth and biomass of winter wheat plants in pot-experiment.

Treatment	Height of Plant (cm)	Root Length (cm)	Dry biomass of Aerial Part of Plant (g)	Dry Biomass of Roots (g)
Control 1	15.1	17.0	0.11	0.12
BZR 337[1]	13.9*	14.7*	0.09	0.11
BZR 348	14.5	16.6	0.10	0.12
BZR 367	14.5	17.1	0.10	0.11
BZR 413	15.1	17.3	0.13	0.12
BZR 416	14.6	17.5	0.11	0.12
BZR 417	14.7	16.9	0.09	0.11
BZR 430	13.6*	14.1*	0.09*	0.10
Control 2	14.6	18.3	0.11	0.15
BZR 441	14.8	16.4*	0.11	0.13
BZR 455	14.4	16.6*	0.09	0.12
BZR 462	14.8	17.6	0.11	0.14
BZR 472	14.6	15.8*	0.12	0.13
BZR 480	15.0	15.5*	0.12	0.14
BZR 512	14.5	16.0*	0.10	0.11*
BZR 538	13.8*	17.7	0.11	0.14
Control 3	13.7	13.0	0.11	0.09
BZR 436	13.6	16.0*	0.14*	0.10
BZR 517	14.1	15.6*	0.12	0.10
Control 4	14.8	15.5	0.10	0.11
BZR 519	15.2	15.7	0.11	0.12
BZR 528	15.8*	15.9	0.12	0.14*
BZR 873	15.6*	15.6	0.11	0.12
Control 5	14.2	16.2	0.10	0.12
BZR 18	15.7*	14.8	0.12	0.12
BZR 59	15.3*	12.9*	0.12	0.10
BZR 658	15.1*	11.5*	0.12	0.10
BZR 673	15.0	15.8	0.11	0.14
Control 6	14.9	14.2	0.11	0.12
BZR 623	15.8*	14.5	0.12	0.14
Control 7	14.8	14.6	0.11	0.10
BZR 86	15.1	14.5	0.11	0.13
BZR 148	14.8	16.5*	0.11	0.13
BZR 187	15.0	14.1	0.12	0.13
BZR 241	15.5	16.6*	0.12	0.15*
BZR 261	14.7	17.0*	0.11	0.13
BZR 277	14.9	13.7	0.11	0.12
BZR 523-1	15.0	17.8*	0.11	0.15*
BZR 523-2	14.8	17.6*	0.12	0.14
Control 8	15.7	15.0	0.13	0.13
BZR 854	14.9*	14.7	0.12	0.13
BZR 862	19.1*	17.2*	0.15*	0.12

[1] Accession number of bacteria in the Bioresource Collection of FSCBPP; *The marked variants are significantly different from the control (according to Duncan's Multiple Range Test, P=0.05, n_{height}=80, n_{length}=80, $n_{dry\ biomass}$=3).
